# The role of toll-like receptor variants in acute anterior uveitis

**Published:** 2011-11-16

**Authors:** Divya S. Pratap, Lyndell L. Lim, Jie Jin Wang, David A. Mackey, Lisa S. Kearns, Richard J. Stawell, Wellcome Trust Case Control Consortium 2, Kathryn P. Burdon, Paul Mitchell, Jamie E. Craig, Anthony J. Hall, Alex W. Hewitt

**Affiliations:** 1 Centre for Eye Research Australia, University of Melbourne, Royal Victorian Eye and Ear Hospital, Melbourne, Australia; 2 Centre for Vision Research, Department of Ophthalmology and Westmead Millennium Institute, University of Sydney, Westmead, Australia; 3 Lions Eye Institute, University of Western Australia, Centre for Ophthalmology and Visual Science, Perth, Australia; 4 Department of Ophthalmology, Flinders University, Flinders Medical Centre, Adelaide, Australia; 5 Department of Ophthalmology, Alfred Hospital, Monash University, Melbourne, Australia

## Abstract

**Purpose:**

Acute anterior uveitis (AAU) is the most common form of uveitis; however, while it is presumed to have an immunological basis, the precise underlying etiology remains elusive. Toll-like receptors (*TLR*s) have a key role in linking innate and adaptive immunity, thereby forming a molecular bridge between microbial triggers and the development of AAU. The purpose of this study was to investigate the role of *TLR2* and *TLR4* gene polymorphisms in the pathogenesis of AAU.

**Methods:**

The study comprised 225 confirmed cases of idiopathic or human leukocyte antigen (HLA) B27 (subtypes B*2701-2759; *HLA-B27*)-related AAU and 2,534 population-based controls from the Blue Mountains Eye Study. All participants were of Anglo-Celtic descent. Blood samples were collected for DNA extraction and genotyping. A total of 16 single nucleotide polymorphisms (SNPs) were selected for analysis and either directly genotyped or imputed to cover the common variations within the *TLR* genes. Data were analyzed at the allelic, genotypic and haplotypic levels.

**Results:**

Control subjects were significantly older than case subjects (p<0.0001). There was no significant difference in the gender composition between the case and control cohorts (p=0.18). One *TLR2* SNP (rs11938228) was found to be associated with AAU at the allelic level (OR=1.28; p=0.017); however this association did not remain following adjustment for age and sex (p=0.067). None of the SNPs at the *TLR4* locus were found to differ significantly between cases or controls, irrespective of adjustment for age and gender.

**Conclusions:**

This study has confirmed that common *TLR* variants of moderate effect size do not predispose to AAU, undermining the implication of reported mutations in the selective perturbations of *TLR* expression and function evident in AAU.

## Introduction

Acute anterior uveitis (AAU) is the most common type of uveitis. It is a leading cause of blindness and significant cause of visual impairment in western populations [1-4]. Although Human leukocyte antigen (HLA) B27 (subtypes B*2701-2759; *HLA-B27*)-related AAU is the most common identifiable form of AAU, approximately 50% of cases are idiopathic with a presumed immune pathogenesis [5,6]. Other genetic and environmental factors are involved in triggering and promoting even *HLA-B27*-associated AAU.

There is a great deal of controversy surrounding the role of infectious agents in non-infectious uveitis. Irrespective of the *HLA-B27* status, microbial triggers, especially Gram-negative enterobacteria such as *Klebsiella, Shigella*, and *Yersinia*, have been implicated in the pathogenesis of AAU [7,8] and related syndromes such as reactive arthritis. These findings suggest that interactions between microbes and Toll-like receptors (*TLR*s) may overcome the protective state of ocular immune privilege leading to intraocular inflammation. The uvea is highly sensitive to lipopolysaccharide, and antigen-presenting cells (APCs) in the iris and ciliary body have been shown to express functional *TLR4*, through which microbial triggers could initiate AAU [9-11]. Furthermore, *TLR*-expressing APCs form a crucial link between the innate and adaptive arms of the immune response. APC stimulation via the *TLR*s results in production of proinflammatory cytokines, which then activate and prime antigen-specific naive T cells, eventually triggering adaptive immunity [10]. Recently, TLRs have been found to have gene-specific regulatory mechanisms, which allow for variation and diversity in immune-pathway regulation [12]. Specifically, *TLR* chromatin modifications are associated with transient silencing of pro-inflammatory mediators, and priming of other genes, which include antimicrobial effectors [12].

The role of infectious agents, particularly gram-negative bacteria, in both human uveitis and experimental uveitis is clear. It is plausible that *TLR*s expressed in the eye trigger innate and then adaptive immunity in response to these common infectious agents or pathogen-associated molecular patterns. This immune response may then manifest as intraocular inflammation characteristic of AAU. We proposed that subtle differences in TLR protein expression (encoded for by various polymorphisms in *TLR* genes) may lead to an increased risk of AAU. In this study, we sought to definitively determine whether common *TLR* variants predispose to AAU in a Caucasian population.

## Methods

### Subject recruitment

Ethical approval for this work was provided by the Human Research & Ethics Committee of the Royal Victorian Eye and Ear Hospital (RVEEH, Melbourne, Victoria, Australia). Approval for the Blue Mountains Eye Study (BMES) was obtained from the relevant Human Research Ethics Committees of the University of Sydney and Western Sydney Area Health Service (Sydney, Australia). This study was conducted in accordance with the Declaration of Helsinki, with signed informed consent obtained from participants at each visit of the study.

Patients with anterior uveitis were recruited from the RVEEH and local private ophthalmology practices. As per the inclusion criteria, participants were required to be Caucasian, aged at least 18 years, able to provide informed consent, and diagnosed by an ophthalmologist as has having either either idiopathic or *HLA-B27*-related anterior uveitis. Participants were excluded if an underlying cause for their AAU had been diagnosed. These conditions included Behçets syndrome, Fuchs heterochromic cyclitis, herpes simplex, reactive arthritis, sarcoidosis, syphilis, sympathetic ophthalmia, herpes zoster, and juvenile idiopathic arthritis.

Control subjects were derived from participants of the BMES, a population-based cohort study of older Australians aged 49 or older living in two postcode areas in western Sydney. This is an ethnically homogenous population of Anglo-Celtic descendants. Control subjects of this study were drawn from participants attending the second cross-sectional survey of the BMES conducted between 1997 and 2001 [13].

### Single nucleotide polymorphism (SNP) selection and genotyping

Data from The International HapMap Project were used to select of a total of 16 SNPs that captures most of the genetic diversity across the *TLR2* and *TLR4* loci. To match the data used for imputation in BMES (see below), data were sourced from the National Center for Biotechnology Information Build 36 (HapMap Data Phase III/Rel#2, Feb09, on NCBI36 B36 assembly, dbSNP b126). SNPs were selected from the Centre d’Etude du Polymorphisme Humain from Utah population of the HapMap project, using pairwise tagging, with an r^2^ threshold set to 0.8 and minor allele frequency (MAF) of 5%. The nine tagging SNPs covering the *TLR*2 locus included: rs13150331 (G/A), rs7696323 (T/C), rs1898830 (G/A), rs1816702 (T/C), rs4696483 (T/C), rs11938228 (A/C), rs3804099 (C/T), rs3804100 (C/T), and rs7656411 (G/T). Seven SNPs were required to tag *TLR*4: rs1927914 (C/T), rs1927911 (T/C), rs11536878 (A/C), rs5030717 (G/A), rs5030728 (A/G), rs1554973 (C/T), and rs7846989 (C/T). To adjust for *HLA-B27* status tagging SNPs across the *HLA-B* locus were also selected (rs2523612; rs2596501; rs9266095; rs2523605; rs2596472; rs2395029; rs2284178; and rs3094014).

Genomic DNA from case participants was extracted from peripheral nucleated blood cells using a standard salting out procedure at the Western Australian DNA Bank. Case genotyping was performed using the iPLEX Gold chemistry (Sequenom Inc., San Diego, California) on an Autoflex mass spectrometer at the Australian Genome Research Facility, Brisbane, Australia.

Participants of the BMES (Cross-section II) who had DNA available were genotyped (n=2,761) using the Illumina Human 670-Quadv1 genotyping array at the Wellcome Trust Centre for Human Genetics, Sanger Institute, Cambridge, England (Appendix 1). Imputation was performed using the 1000 genomes data set (NCBI Build 36.1) as the imputation backbone, using only SNPs passing quality control (minor allele frequency [MAF] >0.01, genotyping call rate >0.95, Hardy–Weinberg p-value >1×10^−6^). Genotype imputation was performed for autosomal SNPs using MACH v1.0.16. Imputed genotypes were excluded if their ratio of observed to expected variance of imputed allele dosages (R^2^) <0.3 or a MAF<0.01 or if they had a Hardy–Weinberg Equilibrium p<1×10^−6^. Four SNPs at the *TLR2* locus (rs13150331, rs1816702, rs3804099, and rs7656411), two SNPs at the *TLR4* (rs1927914 and rs1554973) locus, and all SNPs at the *HLA-B* locus were directly genotyped in the BMES cohort.

### Statistical analysis and power calculations

In Stata/SEM 10.0 (StataCorp, College Station, TX), a Student *t*-test and χ^2^ test was used to compare differences in age and gender, respectively, between cases and controls.

PLINK version 1.06 was used for analysis of genetic data [14]. Allelic, genotypic (dominant, recessive and co-dominant) and haplotypic models were constructed for both loci. Odds ratios (ORs) and 95% confidence intervals (CI) were also calculated. Haplotypes were constructed based on linkage disequilibrium block structure, as defined by Gabriel and colleagues [15]. The logistic regression model for the association between each SNP and the occurrence of AAU incorporated both sex and age as covariates. Additional models also adjusted for haplotypes across the *HLA-B* locus. A value less than 0.05 was considered statistically significant following Bonferroni correction for multiple-test correction. Power calculations were performed using PGA [16]. At the p=0.05 significance level, this study had a power of 80% to detect a disease-associated allele conferring a relative risk ≥1.5 for variants with a frequency >0.1. For variants that had a minor allele frequency >0.05, this study had 80% power to detect variants with a relative risk of 2 at the p=0.05 level.

## Results

Genotyping data were available for 2,759 participants (225 cases and 2,534 population-based controls). Participants were aged between 18 and 92 years (mean=62 years; SD=9.82, 95%CI=61.72–62.46). Controls were significantly older than cases (p<0.0001). There was no significant difference in the gender composition between the case and control cohorts (p=0.18); approximately 57% of the subjects were female. Demographics of the study cohort are presented in Table 1.

**Table 1:** Demographic features of study cohort.

**Characteristic**	**Cases**	**Controls**	**p value**
Number	225	2534	
Number female (%)	118 (52%)	1446 (57%)	0.18
Mean age (SD; 95%CI)	50 (15.7; 47.9 - 52.1)	63 (8.3; 62.8–63–5)	<0.0001

All variants at the *TLR2* locus were within the quality control (QC) parameters. The allele frequencies of nine *TLR2* SNPs in AAU cases and healthy controls are displayed in Table 2, in conjunction with the allelic association results. One SNP (rs11938228) was found to be nominally associated with AAU (p=0.017, before Bonferroni adjustment). The crude OR for developing AAU and having the A allele at this SNP was 1.28 (95% CI: 1.04–1.56). However, this variant was found not to be significant following age- and gender-adjustment (p=0.067). The SNP rs11938228 that was found to be statistically significant at the allelic level remained significant under the co-dominant genotypic level only before adjustment for age and sex (p=0.032). This SNP reached a nominal level of statistical significance under the dominant genetic model following adjustment for age and sex (p=0.045, Table 3). However, the significance of this association was not retained following Bonferroni correction. None of the nine SNPs were implicated under a recessive model before or following age- and gender-adjustment (Table 3).

**Table 2:** Allelic Association at the *TLR2* locus on chromosome 4q31.

** **	** **	** **	** **	** **	**Unadjusted**		** **	** **	Adjusted*		** **
**SNP**	**Minor allele**	**f_case_**	**f_control_**	**p value**	**OR**	**(95% CI)**	**χ^2^**	**p value**	**OR**	**(95% CI)**	**t-stat**
rs13150331	G	0.44	0.41	0.216	1.13	(0.93–1.38)	1.53	0.650	1.05	(0.85–1.31)	0.45
rs7696323	T	0.32	0.32	0.690	0.96	(0.78–1.18)	0.16	0.927	0.99	(0.79–1.25)	−0.09
rs1898830	G	0.38	0.35	0.208	1.14	(0.93–1.39)	1.59	0.293	1.13	(0.90–1.40)	1.05
rs1816702	T	0.11	0.11	0.673	1.07	(0.79–1.45)	0.18	0.652	0.92	(0.65–1.31)	−0.45
rs4696483	T	0.13	0.11	0.099	1.27	(0.96–1.70)	2.72	0.513	1.12	(0.80–1.56)	0.65
rs11938228	A	0.40	0.34	0.017	1.28	(1.04–1.56)	5.72	0.067	1.23	(0.99–1.53)	1.83
rs3804099	C	0.41	0.43	0.420	0.92	(0.76–1.12)	0.65	0.659	0.95	(0.77–1.18)	−0.44
rs3804100	C	0.05	0.07	0.133	0.71	(0.46–1.11)	2.26	0.342	0.80	(0.50–1.27)	−0.95
rs7656411	G	0.24	0.26	0.243	0.87	(0.69–1.10)	1.37	0.654	0.94	(0.74–1.21)	−0.45

Abbreviations: f, frequency; OR, odds ratios; 95%CI, 95% confidence interval; t-stat, t-statistic. *Adjusted for age and sex.

**Table 3:** Genotypic association at the *TLR2* locus on chromosome 4q31 adjusted for age and sex.

** **	**Co-dominant**			**Dominant**			**Recessive**		
**SNP**	**p value**	**OR**	**(95% CI)**	**p value**	**OR**	**(95% CI)**	**p value**	**OR**	**(95% CI)**
rs13150331	0.669	1.05	(0.84–1.31)	0.671	1.07	(0.78–1.49)	0.761	1.06	(0.72–1.58)
rs7696323	0.525	0.92	(0.70–1.20)	0.647	1.08	(0.79–1.47)	0.362	0.78	(0.47–1.32)
rs1898830	0.605	1.07	(0.84–1.36)	0.129	1.28	(0.93–1.75)	0.942	0.98	(0.63–1.55)
rs1816702	0.229	0.48	(0.14–1.59)	0.854	0.97	(0.66–1.40)	0.228	0.23	(0.02–2.53)
rs4696483	0.753	1.12	(0.55–2.26)	0.526	1.12	(0.78–1.61)	0.78	1.22	(0.30–4.96)
rs11938228	0.151	1.19	(0.94–1.51)	0.045	1.39	(1.01–1.91)	0.419	1.20	(0.77–1.85)
rs3804099	0.443	0.91	(0.72–1.15)	0.622	1.09	(0.78–1.51)	0.169	0.75	(0.50–1.13)
rs3804100	0.613	0.76	(0.27–2.17)	0.362	0.79	(0.48–1.31)	0.629	0.60	(0.07–4.83)
rs7656411	0.889	0.98	(0.71–1.34)	0.585	0.92	(0.67–1.25)	0.978	0.99	(0.53–1.84)

Abbreviations: OR, odds ratios; 95%CI, 95% confidence interval.

**Figure 1:**
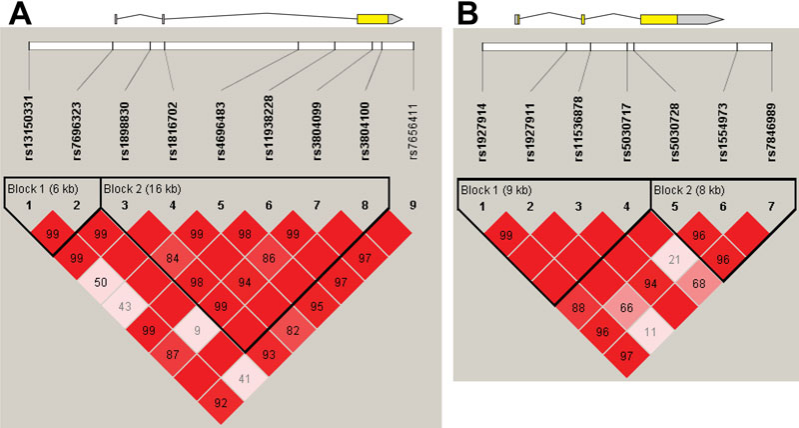
Linkage disequilibrium at toll-like receptor (TLR) loci. **A**: *TLR2*; **B**: *TLR4*.

At the *TLR2* locus, two haplotype blocks were identified under the “solid spine” block definition, as displayed in Figure 1. Block 1 was defined by SNP pair, rs13150331 and rs7696323 and block 2 comprises all SNPs between rs1898830 and rs3804100. There was no significant difference in the frequency of any of the common haplotypes between the case subjects and control participants in block 1 or block 2. Following adjustment for age and gender, one haplotype in block 2 (ACCCTT) was found to occur more frequently in control subjects compared to cases (21.0% vs 17.3% respectively, p=0.031); however, this did not survive Bonferonni correction.

All *TLR4* variants were found to pass QC. The frequencies of seven *TLR4* SNPs in AAU cases and healthy controls are displayed in Table 4, along with the results from allelic association. None of the SNPs were found to differ significantly between cases or controls, irrespective of the adjustment for age and gender. Additionally, no significant association was identified under a co-dominant or recessive genotypic model (Table 5). Under the dominant model, rs1927911 was the only SNP that approached significance, revealing a nominal association with AAU following age and sex-adjusted (Table 5).

**Table 4:** Allelic association at the *TLR4* locus on chromosome 9q33.

** **	** **	** **	** **	** **	**Unadjusted**		** **	** **	Adjusted*		** **
**SNP**	**Minor Allele**	**f_case_**	**f_control_**	**p value**	**OR**	**(95% CI)**	**χ^2^**	**p value**	**OR**	**(95% CI)**	**t-stat**
rs1927914	C	0.31	0.33	0.250	0.88	(0.72–1.09)	1.32	0.281	0.88	(0.69–1.11)	−1.08
rs1927911	T	0.22	0.26	0.093	0.82	(0.65–1.03)	2.82	0.068	0.78	(0.60–1.02)	−1.83
rs11536878	A	0.10	0.12	0.156	0.79	(0.57–1.09)	2.01	0.314	0.84	(0.59–1.19)	−1.01
rs5030717	G	0.10	0.09	0.719	1.06	(0.77–1.47)	0.13	0.709	0.93	(0.64–1.35)	−0.37
rs5030728	A	0.31	0.30	0.462	1.08	(0.88–1.33)	0.54	0.494	1.09	(0.86–1.37)	0.68
rs1554973	C	0.24	0.26	0.216	0.87	(0.69–1.09)	1.53	0.585	0.93	(0.72–1.20)	−0.55
rs7846989	C	0.10	0.10	0.830	1.04	(0.75–1.43)	0.05	0.387	1.17	(0.82–1.67)	0.87

Abbreviations: f, frequency; OR, odds ratios; 95%CI, 95% confidence interval; t-stat, t-statistic. *Adjusted for age and sex.

**Table 5:** Genotypic association at the *TLR4* locus on chromosome 9q33 adjusted for age and sex.

** **	**Co-Dominant**			**Dominant**			**Recessive**		
**SNP**	**p value**	**OR**	**(95% CI)**	**p value**	**OR**	**(95% CI)**	**p value**	**OR**	**(95% CI)**
rs1927914	0.596	0.93	(0.72–1.21)	0.177	0.81	(0.60–1.10)	0.901	0.97	(0.59–1.60)
rs1927911	0.312	0.84	(0.59–1.18)	0.056	0.74	(0.54–1.01)	0.489	0.79	(0.40–1.55)
rs11536878	0.894	1.04	(0.55–1.97)	0.247	0.8	(0.54–1.17)	0.832	1.15	(0.32–4.07)
rs5030717	0.526	1.24	(0.64–2.40)	0.557	0.88	(0.59–1.33)	0.499	1.58	(0.42–5.90)
rs5030728	0.26	1.16	(0.90–1.50)	0.862	1.03	(0.76–1.40)	0.214	1.34	(0.83–2.25)
rs1554973	0.973	1.01	(0.74–1.38)	0.433	0.88	(0.65–1.20)	0.823	1.07	(0.58–1.98)
rs7846989	0.811	0.88	(0.32–2.46)	0.329	1.21	(0.83–1.76)	0.782	0.75	(0.10–5.83)

Abbreviations: OR, odds ratios; 95%CI, 95% confidence interval.

On investigating the haplotype associations at the *TLR4* locus, two blocks were identified under the “solid spine” block definition, as displayed in Figure 1. Block 1 was defined by all SNPs between rs1927914 and rs5030717 and block 2 as SNPs rs5030728 to rs7846989. None of the imputed haplotypes were found to differ significantly between the case and control cohorts.

Three SNPs across the *HLA-B* locus (rs2523612; rs9266095; and rs2523605) were found to be out of Hardy–Weinberg Equilibrium and were subsequently excluded from analysis. There was no significant change in *TLR3* or *TLR4* allelic or genotypic results following adjustment for *HLA-B* haplotypes (data not shown).

## Discussion

The proteins encoded by *TLR2* and *TLR4* are phylogenetically conserved pattern recognition receptors of immune cells like macrophages and neutrophils that have a fundamental role in activation of innate immunity [17,18]. They are one of the first lines of host defense and recognize pathogen-associated molecular patterns associated with various infectious agents [17]. This in turn initiates the proinflammatory cytokine cascade necessary for development of effective immunity through the transcription factor, nuclear factor-kappa B pathway [18]. TLR2 binds various lipoproteins, including gram-positive bacterial components such as peptidoglycan [10]. TLR4 has been implicated in signal transduction events induced by lipopolysaccharide found in the majority of gram-negative bacteria, which is pivotal in the pathogenesis of some cases of non-infectious immune-mediated AAU [8,10].

Despite an observed difference in *TLR* expression and function [9,19] in patients with active AAU, our study found no statistically significant association between common SNPs at the *TLR2* or *TLR4* loci and AAU. Given the vital role *TLR*s have in both the innate and adaptive immune systems [20], our results suggest that changes in *TLR* expression and function, not polymorphisms, may be associated with disease development. Given that the *TLR*s are involved in various inflammatory diseases such as inflammatory bowel disease, rheumatoid arthritis and psoriasis, all of which are recognized systemic associations of *HLA-B27* AAU [21,22], our findings may also indicate that the underlying pathogenic mechanism in isolated AAU differs from *HLA-B27* AAU.

Chang and colleagues [19,23] reported a significant reduction in the levels of *TLR2* expression in patients with active AAU as a result of *TLR* activation by gram-negative and/or gram-positive bacterial products, for example lipopolysaccharides or peptidoglycans, respectively. Conversely, selective functional hyporesponsiveness to *TLR4* stimulation was demonstrated, wherein, cytokine production was markedly reduced [19]. Given that the patients had active AAU at the time of testing, the lipopolysaccharide hyporesponsiveness was consistent with a functional state of endotoxin tolerance induced by recent exposure to lipopolysaccharide [19,24]. In a preliminary investigation in the genetic contribution of *TLR2* and *TLR4*, Chang and colleagues genotyped the Arg753Gln (rs5743708) *TLR2* and Asp299Gly *TLR4* variants in nine patients with AAU. Although no statistically significant difference was identified, this previous work was underpowered and would fail to identify other exonic or cis-acting variants, which may alter gene expression. Interestingly, despite identifying an increased expression of *TLR3* and *TLR4* in buccal mucosa of patients with Behçet's disease, Durrani and colleagues failed to identify any significantly associated SNP in these genes [25].

Intraperitoneal administration of endotoxin or lipopolysaccharide gives rise to an endotoxin-induced uveitis (EIU) in rodent and is one of the best animal models of human AAU [26,27]. Interestingly, there is variation in rodent susceptibility to EIU, which may reflect the importance of genetic and environmental factors in disease predisposition in humans. In EIU, the lipopolysaccharide hyporesponsiveness observed in certain mice can be attributed to a point mutation located in the coding region of the *TLR4* gene, consequently disrupting downstream signal transduction [10,28]. However, our results do not support this EIU evidence which has indicated a transient state of hyporesponsiveness to *TLR4* stimulation induced by a pre-exposure to lipopolysaccharide in lieu of functional *TLR4* gene mutations.

Strengths of our study include that it was based on a biologically strong and plausible hypothesis, with a large sample size of 2,759 participants (225 cases and 2,534 controls) and reasonable power to detect a common variant of modest effect size. Although imputation is a boon in the context of producing a powerful study design, it can be a bane in the absence of strong linkage disequilibrium. Fortunately, despite using imputed data in control participants, we had excellent call rates (>95%) and imputation parameters at both loci. Our use of historic control data did not allow us to directly determine *HLA-B27* carrier status, rather only SNP genotypes and haplotypes across the *HLA-B* gene could be analyzed.

In summary, we undertook a large case-control study to investigate the influence of *TLR* polymorphisms on predisposition to AAU. We found no strong evidence for common *TLR2* or *TLR4* variants and idiopathic AAU in the Caucasian population. Clearly, further work investigating possible trans-acting *TLR* variants that alter gene expression and function should be performed. The quest for etiological factors other than *HLA-B27* is fundamental for monitoring disease onset and progression. Eventually we hope to target specific steps in the pathogenic pathway, potentially revolutionising therapeutic regimens for this sight-threatening ocular condition.

## Appendix 1. Membership of the Wellcome Trust Case Control Consortium 2 and of The Blue Mountains Eye Study GWAS Team.

To access the data, click or select the words “Appendix 1.” This will initiate the download of a compressed (pdf) archive that contains the file.
